# Research and Remedies

**Published:** 1913-05-03

**Authors:** 


### RESEARCH AND REMEDIES.

### How Typhoid is Spread.

If there are still any sceptics who doubt the reality of the " typhoid carrier " and the influence which such individuals exercise in the propagation of this disease, they can be recommended to study the remarkable case published by Bull and Kidd in the *Australian Medical Journal*. In 1895 a woman of sixty had an attack of typhoid fever and recovered. She got her infection from a son, whom she nursed: he soon afterwards left the district, and could therefore not be to blame for any further cases. She assisted in the management of a small wayside hotel in a country district, containing a scattered population of some 1,500 persons. It was this old lady's duty to attend to the cows, and it is significant that she always refused to have the milk boiled. In 1898 another son got typhoid, from which he died. Thirteen more cases occurred during the next thirteen years. Five of the patients were boarders in the hotel, and two were tourists who had stayed there. Three were neighbours supplied with milk from the hotel cows; two were servants in the hotel; and the thirteenth was the the old lady's niece. Then in 1911 the " carrier " removed to another hotel in a different district: no further cases occurred in the first district after she left it, but five cases promptly appeared in the second hotel. Just as preparations for investigation were being considered, the old lady died. Her gall bladder was removed two hours after death, and yielded vast numbers of typhoid bacilli: the intestines, however, showed bacillus coli communis, but no bacillus typhosus. The chain of evidence seems strong, and the fact that the " typhoid carrier " continued to disseminate virulent germs up to her death at the age of seventy-six is also remarkable.

### Spinal Anæsthesia in Childhood.

In the *West London Medical Journal* Mr. Tyrrell Gray pleads strongly for spinal anæsthesia in operations for the acute peritoneal affections of children. He holds that in adults spinal anæsthesia is advisable in very rare instances alone; but that it is imperatively called for when dealing with an " acute abdomen " in a young subject. The considerations which lead him to this pronouncement are somewhat complicated, and depend upon the relations of " nerve-blocking " to shock and " shock-value." Mr. Gray does not enter into the merits of the Crile-Henderson controversy as to the causation of shock. But his practice is evidently nearer to that of Henderson than of Crile; for he condemns vaso-constrictors and praises strychnine very highly. The latter drug is recommended for constant employment on the ground that it " reopens conduction across synapses." This may or may not be the case, and must be exceedingly difficult to prove; we would rather see some more practical explanation offered, or better still none at all in the present imperfect state of knowledge. The true criterion is: Does strychnine do good or not? It may be added that he gives morphine with atropine freely, and does not attempt to move the bowels at all, preferring to leave them to act naturally.

